# Prenatal hypoxia inhibited propionate‐evoked BK channels of mesenteric artery smooth muscle cells in offspring

**DOI:** 10.1111/jcmm.14994

**Published:** 2020-01-24

**Authors:** Wenna Zhang, Xueqin Feng, Yumeng Zhang, Miao Sun, Lingjun Li, Qinqin Gao, Jiaqi Tang, Pengjie Zhang, Juanxiu Lv, Xiuwen Zhou, Zhice Xu

**Affiliations:** ^1^ Institute for Fetology First Hospital of Soochow University Suzhou China

**Keywords:** large‐conductance calcium‐activated potassium channels, offspring, prenatal hypoxia, propionate

## Abstract

As a common complication of pregnancy, gestational hypoxia has been shown to predispose offspring to vascular dysfunction. Propionate, one of short‐chain fatty acids, exerts cardioprotective effects via reducing blood pressure. This study examined whether prenatal hypoxia impaired propionate‐stimulated large‐conductance Ca^2+^‐activated K^+^ (BK) channel activities in vascular smooth muscle cells (VSMCs) of offspring. Pregnant rats were exposed to hypoxia (10.5% oxygen) and normoxia (21% oxygen) from gestational day 7‐21. At 6 weeks of age, VSMCs in mesenteric arteries of offspring were analysed for BK channel functions and gene expressions. It was shown firstly that propionate could open significantly BK single channel in VSMCs in a concentration‐dependent manner. Antagonists of G protein βγ subunits and inositol trisphosphate receptor could completely suppress the activation of BK by propionate, respectively. Gα_i/o_ and ryanodine receptor were found to participate in the stimulation on BK. Compared to the control, vasodilation and increments of BK NPo (the open probability) evoked by propionate were weakened in the offspring by prenatal hypoxia with down‐regulated Gβγ and PLCβ. It was indicated that prenatal hypoxia inhibited propionate‐stimulated BK activities in mesenteric VSMCs of offspring via reducing expressions of Gβγ and PLCβ, in which endoplasmic reticulum calcium release might be involved.

## 1 INTRODUCTION

Gestational hypoxia is a common factor of various complications in pregnancy, such as pre‐eclampsia, anaemia, gestational diabetes or high‐altitude pregnancy.^1^ The foetus adapts to hypoxaemia at the cost of modifying organ system maturation and redistributing blood flow towards essential circulations, consequently leading to intrauterine growth restriction in short term and foetal programming of cardiovascular diseases in long term.^2^

Much of research has examined vasculature alterations in offspring exposed to hypoxia in foetal life. Dysfunctions of endothelium‐dependent vasodilatation by maternal hypoxia have been demonstrated in middle cerebral arteries,^2^ mesenteric arteries,^3^, ^4^, ^5^, ^6^ renal arteries 7, 8 and femoral arteries.^9^, ^10^ More studies described increased blood pressure responses and greater vasoconstrictions of resistance arteries in the offspring exposed to prenatal hypoxia,^11^, ^12^, ^13^ which were associated with altered vascular constrictor and dilator mechanisms. Among underlying factors are calcium and potassium channels, which play pivotal keys in contraction and hyper‐polarization of vascular smooth muscle cells (VSMCs),^7^, ^11^, ^12^, ^14^ respectively. Nevertheless, the detailed molecular mechanisms through which hypoxia in utero affects vascular functions of foetuses and offspring have yet to be elucidated.

Short‐chain fatty acids (SCFAs) are gut microbial metabolites produced by fermentation of dietary fibres, mainly including acetate, propionate and butyrate.^15^ Beyond the important physiological roles of shaping the gut environment and energy homeostasis, SCFAs are known to regulate several cellular processes in other tissues by activating membrane receptors (Gpr41, Olr59, Ffar2 and so on), inhibiting histone deacetylases, and stabilizing the hypoxia‐inducible factor.^16^, ^17^, ^18^ Moreover, the protection of SCFAs against hypertension has been demonstrated recently in different models. On one hand, SCFAs reduce risk factors of cardiovascular diseases, including obesity by improving appetite regulation^19^ and type 2 diabetes mellitus by improving insulin sensitivity.^20^ On the other hand, SCFAs could regulate blood pressure by relaxing resistance arteries^21^, ^22^, ^23^ and releasing renin.^24^ In addition, the anti‐hypertensive effect of propionate has been proposed to be linked with anti‐inflammatory properties via regulating T helper cell homeostasis.^25^ In spite of uncovering the beneficial effects of SCFAs in various ways, understanding of the intracellular mode of actions is limited.

Based on the characteristics of SCFAs‐induced vasodilatation, effects of propionate on large‐conductance Ca^2+^‐activated K^+^ (BK) channel activity in VSMCs were investigated in this study for the first time. Downstream signal pathway of propionate receptors was tested to explore the underlying mechanisms. Considering the increased vasoconstrictions in rat offspring suffered from hypoxia in utero, we hypothesized that prenatal hypoxia inhibited propionate‐dependent BK activities in mesenteric arteries of the offspring. To test the hypothesis, basal open probability (NPo) of BK channels, propionate‐stimulated NPo of BK with and without specific antagonists, and expressions of relative proteins in mesenteric VSMCs were compared between prenatal hypoxia offspring and the control. The information obtained extends the physiological functions of propionate and the influence of prenatal hypoxia on BK dysfunction in the offspring.

## 2 MATERIALS AND METHODS

### 2.1 Animals

Sprague‐Dawley rats (female: 300‐350 g, male: 400‐450 g) were purchased from Animal Center of Soochow University and housed in a controlled environment (24 ± 0.2°C) under a 12 light‐dark cycles (8:00 am‐8:00 pm) with standard rat diets. One female rat was caged with two male rats overnight and pregnancy was confirmed by the presence of vaginal plug next day, which was regarded as the first day of gestation. Pregnant rats were randomly divided into two groups (N = 15 each): control (CON) and prenatal hypoxia group (HY). From gestation day 7‐21, HY rats were placed in hypoxia chambers (10.5% O_2_) and the CON rats in normal circumstances (21% O_2_), with free access to food and water. All rats were raised in normal environment after gestational day 21. After delivering, all rats were provided with tap water and standard rat food, and eight pups per litter were culled to receive breastfeeding and maternal care. A 6‐week‐old male offspring with different mothers were killed using sodium pentobarbital (100 mg/kg intraperitoneally) and used for following experiments. It has been reported that there exist sex differences in vascular function of offspring exposed to prenatal hypoxia.^26^, ^27^ In order to get rid of the effects of sex hormones, the focus of the present study was on male offspring. All experimental procedures were approved by the Ethical Committee of First Hospital of Soochow University and carried out conforming to the SSR's specific policies and procedures.

### 2.2 Measurement of vessel tone

Third‐order mesenteric arteries in 6‐week‐old male offspring were separated from connective tissue, cut into 3 mm in length and recorded with multi‐myograph system (Radnoti) in 5 mL modified Krebs' solution (mmol/L: NaCl 115, NaHCO_3_ 25, KCl 4.6, NaH_2_PO_4_ 1.2, MgCl_2_ 1.2, CaCl_2_ 2.5, and glucose 10; pH 7.4 with NaOH), continuously gassed with 95% O_2_‐5% CO_2_. A total of 120 mmol/L KCl was used to achieve maximal tension. After equilibration, cumulative concentration of phenylephrine (PE) was added into chambers and the contractions were normalized to maximal tension. Increasing dose of sodium propionate (SP) was added after application of 60 mmol/L KCl. Changes in tension by SP were expressed as per cent of the contraction induced by 60 mmol/L KCl.

### 2.3 Cell culture

Vascular smooth muscle cells were isolated from mesenteric arteries in 6‐week‐old male rats, as previously mentioned.^28^ Briefly, mesenteric arteries were stripped off adipose and connective tissues in pre‐cooling phosphate‐buffered saline (PBS). First order of mesenteric arteries were opened longitudinally and cut into pieces in a culture dish. Pieces attached to the dish were maintained in Dulbecco's Modified Eagle's Medium with high glucose (Thermo), including 100 U/mL penicillin, 100 mg/mL streptomycin (Thermo) and 10% foetal bovine serum (Bovogen) in the humidified incubator at 37°C with 95% air and 5% CO_2_. The cells would grow out successively from the original mesenteric arteries pieces after approximately three days. All procedures were carried out under sterile conditions in laminar air flow bench. Confirmed as VSMCs using immunofluorescence of α‐smooth muscle actin (α‐SMA), the cells at third‐fifth passages were used for the experiments.

### 2.4 Immunohistochemistry and immunofluorescence

Third‐order mesenteric arteries were isolated and cleared of adipose and connective tissues, followed by immediately fixed in 10% neutral‐buffered formalin solution. Embedded in paraffin, vessel rings were serially sectioned at 6 μm. According to the standard immunohistochemical staining procedures,^29^ Gpr41 and Olr59 were measured in the vessel sections using primary antibody (Gpr41, 1:100, Santa Cruz, USA; Olr59, 1:100, Sigma, USA). Nuclei were visualized with haematoxylin. Negative controls were performed by omission of the primary antibodies.

Vascular smooth muscle cells grown on coverslips were washed by PBS and fixed in 10% neutral‐buffered formalin solution. After incubation with PBST (PBS with 0.5% TritonX‐100) for 15 minutes, slides were blocked for 1 hour. Then, primary antibody of α‐SMA (Beyotime) and TRITC‐conjugated secondary antibody (Sangon) were applied. Nuclei were counterstained by DAPI (Beyotime). Negative controls were performed by omission of the primary antibodies.

### 2.5 Electro‐physiological measurements on cells

Smooth muscle cells from mesenteric arteries were enzymatically dissociated as previously described.^30^ To be specific, the third‐order mesenteric arteries were cut into pieces in ice‐cold Ca^2+^‐free physiological saline solution (PSS), containing (in mmol/L) the following: KCl 5.6, NaCl 137, MgCl_2_ 1, HEPES 10, Na_2_HPO_4_ 0.42, NaH_2_PO_4_ 0.44, NaHCO_3_ 4.2 and glucose 10 (pH 7.4 with NaOH), and then incubated with 4 mg/mL papain (Solarbio), 2 mg/mL bovine serum albumin (Biosharp) and 1 mg/mL dithiothreitol (Biosharp) for 40 minutes at 37°C. Then, tissues were washed three times with ice‐cold Ca^2+^‐free PSS and dispersed to gain single smooth muscle cells. A 200‐μm nylon mesh was used to percolate tissue debris. The cell suspension was stored at 4°C for study within 6 hours.

For cell patch‐clamp experiments, multiclamp 700B amplifier (Axon) was used to measure single‐channel currents in pipette and bath solutions containing (in mmol/L) MgCl_2_ 10, KCl 140, CaCl_2_ 0.1, HEPES 10 and Glucose 30, pH 7.2 with KOH in pipette solution and pH 7.4 with KOH in bath solution. The number of channels in the patch (N) and the channel open probability (Po) were regarded as an index of the channel steady‐state activity. The BK channel activity was achieved using the following equation:

$$\text{NPo}=\Sigma \left(t_{1}+t_{2}\ldots t_{i}\right),$$

where t_i_ is referred as the relative open time (time open/total time) for each level. The basal activities of BK channels per patch were observed when patches were held at + 50 mV in the bath solution containing 10 µmol/L Ca^2+^. Then, propionate‐stimulated BK NPo was recorded after cumulatively increasing concentrations of SP (10 mmol/L, 20 mmol/L, 25 mmol/L and 50 mmol/L) or 25 mmol/L SP were added to the bath solution. Specific antagonists, including 10^‐5^ mol/L pertussis toxin (PTX, Tocris), 10^‐5^ mol/L gallein (Tocris), 10^‐6^ mol/L 2‐aminoethoxydiphenyl borate (2‐APB, Sigma) and 10^‐7^ mol/L ryanodine (Tocris), were applied to the bath prior to the SP for 30 minutes pre‐incubation. Clampfit 10.2 software (Axon Instruments) was used to analyse average channel activity (NPo).

### 2.6 RT‐PCR and Quantitative real‐time PCR

Primary VSMCs attached to dishes were washed by PBS and then lysed using Trizol reagent (Takara) according to manufacturer's instructions. First‐strand cDNA was synthesized using Prime Script^TM^ II 1st Strand cDNA synthesis kit (Takara). RT‐PCR was performed in 50 μL system, containing 25 µL *Taq* PCR mix (Sangon), 2 µL primer mix, 2 µL cDNA and 21 µL nuclease‐free water with the following programme: one cycle at 95°C for 2.5 minutes; 25 cycles at 95°C for 15 seconds, 55°C for 30 seconds, 72°C for 1 minutes and 72°C for 5 minutes. PCR products were detected using agarose gel electrophoresis.

Mesenteric arteries from offspring were homogenized. Total RNA was isolated using Trizol reagent (Takara) according to manufacturer's instructions. The concentration and purity of extracted RNA were identified by spectrophotometer (Bio‐Rad). First‐strand cDNA was synthesized using Prime Script^TM^ II 1st Strand cDNA synthesis kit (Takara, China). The cDNA was diluted in nuclease‐free water and stored at −20°C before testing. Real‐time PCR was carried out in 20 μL system containing 10 µL of SYBR Premix (Takara), 8 μL of nuclease‐free water, 0.5 μL of forward primer (10 μmol/L), 0.5 μL of reverse primer (10 μmol/L) and 1 μL cDNA. The information of primer pairs was shown in Table 1. The PCR was performed using iCycler MyiQ two Color Real‐Time PCR Detection System (Bio‐Rad) with the following programme: one cycle at 95°C for 5 minutes; 40 cycles at 95°C and 60°C for 15 seconds each. Data were calculated using the threshold cycle (Ct) relative quantification method (2^‐ΔΔCt^). Expressions of genes were normalized to the level of β‐actin.

**Table 1:** PCR primer sequences

Gene name	Forward primer sequence	Reverse primer sequence
Gpr41	TTTTCATGGTGCCCCTGTGT	GGGACATATTGTAGGGGCCG
Ffar2	CCCGGTGCAGTACAAGCTAT	CTGCTCGGTTGAGTTCAGGT
Olr59	GGACCTATTTTGAGCTGTGAGG	TCAGCCGCAAGGTTAGTGTT
eNOS	ACTGGTATTGCACCCTTCCG	GTCCTCAGGAGGTCTTGCAC
β‐actin	CACCCGCGAGTACAACCTTC	CCCAACCCACCATCACACC
Kcnma1	ACTTCGCTTCAGGACAAGGA	ATGGGAATGTTGACTCCGGT
Kcnmb1	TCAAGGACCAGGAAGAGCTG	AGGCTGTCTGGTAGTTGTCC
Gnb3	CGTCCGTAGCCTTCTCACTC	AAAGAACGCCTACACGCTCA
Gnb4	CTAAGATCCCTTGCTGCGGT	ACAAGCAAGAGTCTGTCGGG
Gnb5	ACTACCCCAGTGGAGATGCT	AATCCACGCTTGATGCTCCA
Plcb3	CTGCCTGTCTCTGCTATCCG	ACGTGCTTGATGGGGTTGAT
Plcd1	CGTGTCCGGATCATCTCTGG	CACCGTGGGTTGAAACCATT
Plcg1	CAGCAATCCTAGAGCCGGAG	CCGGTTGTTTTTGCCCTCAG
IP3R1	CCTGTTGACCTAGACAGCCA	AGAACATCCACGAGCACAGA
IP3R2	GCAACAACTACCGGATCGTC	AGGAAGGTGTGGGCTAAGTC
IP3R3	CTGACAGAGGAGACCAAGCA	GAACACTGCCAGGTTGAAGG
RyR1	CGCATGACGCCCCTATACAA	CGGCTGAAGTGTAGGACCAA
RyR2	CCTGGGCTCACAACAAGCTA	CTGAACCATCCCAGCGAACT
RyR3	GAATCAGTGAGTTACTGGGCATGG	CTGGTCTCTGAGTTCTCCAAAAGC

### 2.7 Western blot

Mesenteric arteries of offspring were homogenized in liquid nitrogen and lysed in RIPA lysis buffer (Beyotime), containing protease and phosphatase inhibitor cocktails (Biotool). Proteins were separated by 8%‐12% SDS‐polyacrylamide gradient gels and transferred to 0.45 μm PVDF membranes. Blocked with 5% skim milk, the membranes were incubated with primary antibodies of Kcnma1 (1:500, Santa Cruz, USA), Kcnmb1 (1:1000, Immunoway, China), Gpr41 (1:500, Santa Cruz, USA), Olr59 (1:1000, Sigma, USA), Gnb5 (1:1000, Proteintech, China), PLCβ3 (1:1000, Proteintech, China) and β‐actin (1:10 000, Proteintech, China). Blots were detected using enhanced chemiluminescence detection reagents (Advansta), and specific bands were analysed using a Bioimaging System (Tanon). Protein expressions were presented as relative richness normalized to β‐actin.

### 2.8 Statistical analysis

Results were exhibited as mean ± standard error of mean (SEM). Statistical differences were decided by unpaired Student's *t* test with Welch's correction or two‐way ANOVA with Bonferroni post test when appropriate. Data were analysed and curve fitted using GraphPad Prism 5.0 software. A value of *P* (two‐tailed) <.05 was considered significance.

## 3 RESULTS

### 3.1 Prenatal hypoxia increased PE‐induced vasoconstriction and decreased propionate‐induced relaxation in offspring

**Figure 1:**
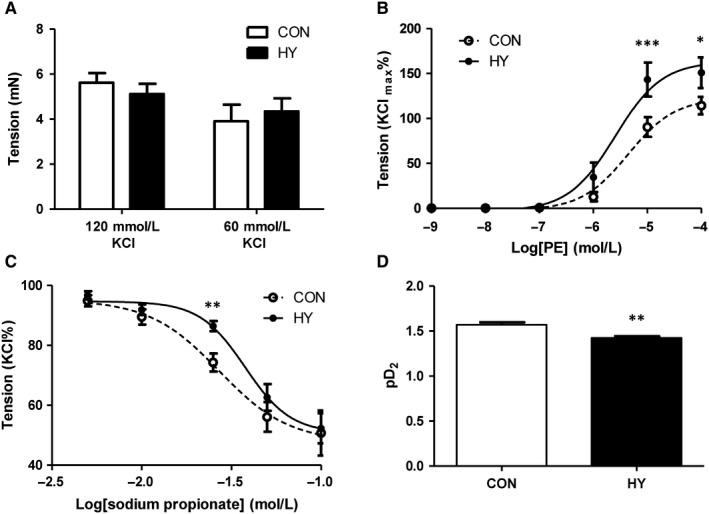
Vessel tone responding to stimulations in offspring mesenteric arteries. A, KCl‐mediated constriction; n = 15 each group (not consanguineous). B, Cumulative concentration of PE‐induced response curves. The responses of PE are presented as the percentage of maximal contraction induced by 120 mmol/L KCl; n = 15 each group (not consanguineous). *P < .05, ^b^P < .001, HY vs CON. C, Dose‐response curves of propionate on 60 mmol/L KCl‐induced contraction; n = 15 each group (not consanguineous). **P < .01, HY vs CON. D, The sensitivity of vasodilation to propionate (pD_2_); n = 15 each group (not consanguineous). **P < .01, HY vs CON

Mesenteric artery was used as a representative of peripheral resistance vessel. Vessel tone responding to PE was tested to evaluate the contraction function of mesenteric artery. There was no significant difference in KCl‐mediated contraction (Figure 1A). According to the concentration‐response curves of PE‐induced vasoconstriction shown in Figure 1B, mesenteric arterial contraction in HY responding to 10^‐5^ and 10^‐4^ mol/L PE was greater than that in CON. Although the maximal relaxation induced by SP was similar between HY and CON, 25 mmol/L SP‐induced vasodilation was weaker in HY than that in CON (Figure 1C), with decreased pD_2_ values (CON: 1.569 ± 0.088, HY: 1.424 ± 0.057, *P* < .01, shown in Figure 1D).

### 3.2 Distribution of SCFAs receptors in mesenteric artery

**Figure 2:**
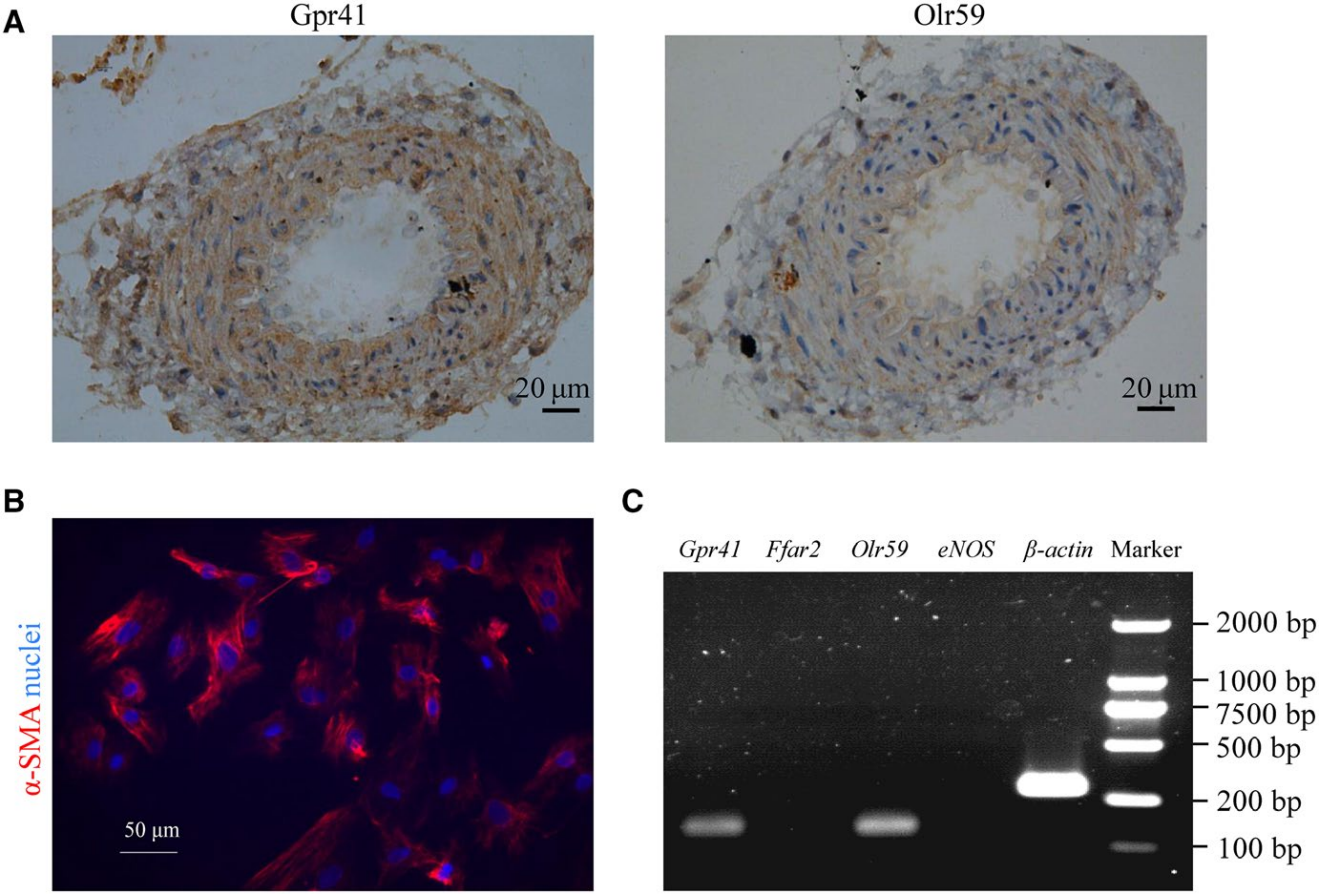
Short‐chain fatty acids receptors are present in rat mesenteric arterial VSMCs. A, Representative immumohistochemistry images of Gpr41 and Olr59 in rat mesenteric arteries (Bar: 20 μm); n = 5. B, Representative immunofluorescence staining images of α‐SMA in primary mesenteric VSMCs. Nuclei were counterstained by DAPI. Bar: 50 μm; n = 5. C, Ethidium bromide‐stained agarose gel of RT‐PCR products amplified from SCFAs receptors in rat mesenteric VSMCs with β‐actin as positive control and eNOS as negative control; n = 5

Short‐chain fatty acids and SCFAs receptors, Gpr41 and Olr59, were identified as novel regulators for blood pressure. In this context, presences of Gpr41 and Olr59 in mesenteric artery of rats were investigated by immunohistochemistry. As shown in Figure 2A, Gper 1 and Olr59 expressed in mesenteric artery. Isolated and cultured mesenteric VSMCs were confirmed positive by immunofluorescent staining of smooth muscle marker, α‐SMA (Figure 2B). *Gpr41* and *Olr59* rather than *Ffar2* were expressed in primary mesenteric VSMCs by RT‐PCR analysis with positive control of *β‐actin* and negative control of *eNOS* (Figure 2C), indicating that VSMCs could respond directly to SCFAs independent on endothelium.

### 3.3 Prenatal hypoxia inhibited propionate‐triggered BK activities in mesenteric VSMCs

**Figure 3:**
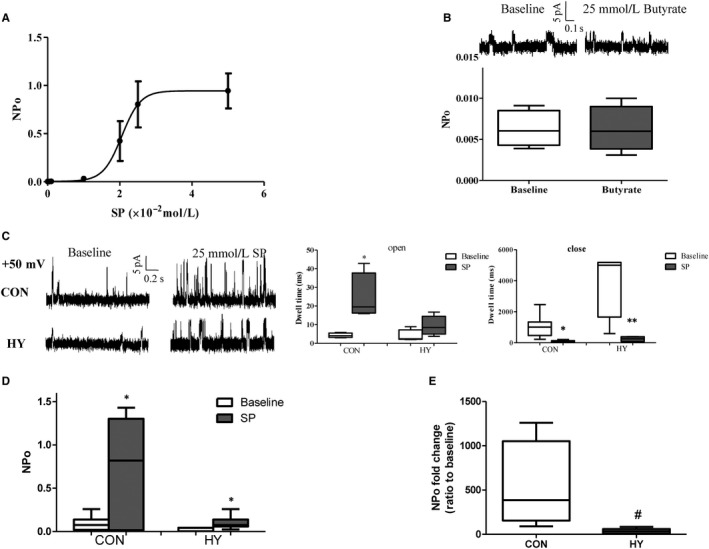
Prenatal hypoxia reduced sensibility of BK to propionate. A, Dose‐response curves for sodium propionate (SP)‐induced single BK channel activities in mesenteric VSMCs. NPo, open probability; n = 10. B, Butyrate failed to trigger the opening of BK; n = 10. C, Representative BK single‐channel current recordings from inside‐out patches and dwell time of open and closed state of BK channels monitored at + 50 mV; n = 15 each group (not consanguineous). *P < .05, **P < .01, SP‐stimulation vs baseline. D, Basal and SP‐stimulated open probability of BK in mesenteric arteries; n = 15 each group (not consanguineous).*P < .05, SP‐stimulation vs baseline. E, NPo of BK stimulated by SP relative to baseline; n = 15 each group (not consanguineous). #, *P* < .05, HY vs CON

It is generally accepted that SCFAs are capable of inducing vasorelaxation in resistance arteries, such as caudal arteries and mesenteric arteries.^22^, ^23^ As a primary potassium channel, BK is involved in hyper‐polarization and subsequent relaxation of VSMCs. Therefore, effects of SCFAs on activities of BK were detected via inside‐out patch‐clamp. At the testing potential of + 50 mV and 10 μmol/L [Ca^2+^]_free_, it was found that SP activated BK single channel in a concentration‐dependent manner (Figure 3A). Twenty‐five mmol/L SP, approximate EC_50_, was used in the subsequent experiments. Figure 3B illustrated that another SCFAs, sodium butyrate, failed to alter the NPo of single BK significantly, indicating different vasorelaxation mechanism independent of BK.

Accumulating studies demonstrated that prenatal hypoxia was linked to developmental origins of cardiovascular diseases in offspring, which were involved in various organs and molecular mechanisms. Based on the finding that prenatal hypoxia induced vasomotor dysfunction of mesenteric arteries in offspring, effects of prenatal hypoxia on SP‐induced BK opening in offspring mesenteric VSMCs were investigated. No significant differences of BK NPo at baseline between CON and HY were observed. In mesenteric VSMCs from CON, the mean open time of single BK channel was profoundly lengthened by SP. In contrast, the mean close time was significantly decreased (Figure 3C). Consequently, the NPo of single BK was elevated significantly in the control offspring (Figure 3D). Compared with CON, there was no difference in the mean open time of single BK from HY before and after stimulation of SP. Albeit with decreased close time and increased NPo by SP, increasing range of NPo in HY was smaller than that in CON (Figure 3E).

### 3.4 Propionate‐dependent mechanism of BK activation

**Figure 4:**
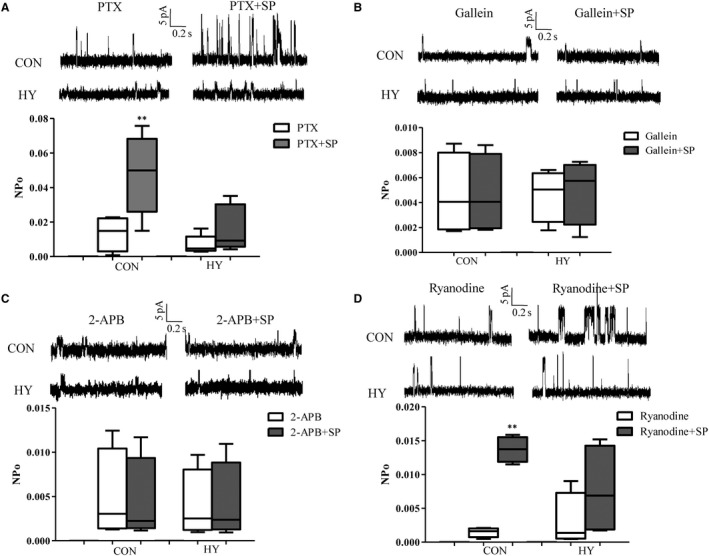
Open probability of BK induced by propionate with pre‐incubation of pertussis toxin (PTX, A), gallein (B), 2‐aminoethoxydiphenyl borate (2‐APB, C) and ryanodine (D); n = 15 each group (not consanguineous). **P < .01, SP‐stimulated BK in the presence of antagonists vs baseline of BK in the presence of antagonists

Propionate receptors, Gpr41 and Olr59, are known to be Gα_i/o_ and Gα_s_ coupled receptors, respectively. Specific blockers were applied to address the mechanisms which underlie propionate‐dependent BK opening. Pre‐incubation of Gα_i/o_ specific inhibitor, PTX, blocked SP‐sensitive BK currents partially (Figure 4A). Without PTX, SP‐evoked BK was 531.4 (NPo) times that at baseline, while SP‐evoked BK with PTX was 21.52 times (*P* < .001; Table 2). G protein β subunit (Gnb) and IP3R‐specific antagonists, gallein and 2‐APB, respectively, inhibited completely SP‐sensitive BK currents (Figure 3B, 3), indicating Gnb and IP3R were indispensable for the stimulation of SP. NPo of BK was still increased by SP after blocking ryanodine receptor (RyR) by ryanodine (Figure 3D), but the amplification was reduced (14.99 with ryanodine, 531.4 without ryanodine, *P* < .001).

**Table 2:** Modulation of propionate‐increased NPo of BK

Inhibitor	The ratio of stimulation to baseline	
	CON	HY
‐	531.4 ± 253.5	35.54 ± 12.93^a^
PTX	21.52 ± 17.06^b^	2.630 ± 0.9771
Gallein	1.075 ± 0.1995^b^	0.8320 ± 0.4559
2‐APB	0.8808 ± 0.1174^b^	0.9523 ± 0.1124
Ryanodine	14.99 ± 8.612^b^	3.578 ± 0.7766

Note

Data are means ± SEM.

a *P* < .001, HY vs CON.

b *P* < .001, propionate‐dependent BK vs propionate‐dependent BK with antagonist.

As for NPo of BK in mesenteric VSMCs from HY in the presence of the four antagonists, there were no significant differences before and after stimulation of SP (Table 2), indicating complete inhibitions. These results suggest that G protein‐IP_3_R/RyR might be involved in cellular mechanisms of SP‐sensitive BK opening.

### 3.5 The effect of prenatal hypoxia on expression of Gnb‐PLC‐IP_3_R/RyR‐BK signalling pathway in the offspring

**Figure 5:**
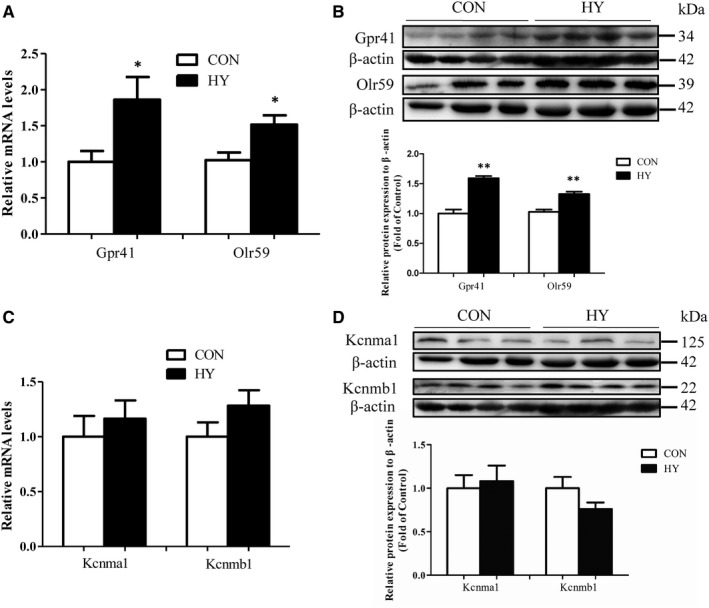
Expressions of SCFA receptors and BK subunits in mesenteric arteries. A, mRNA expressions of Gpr41 and Olr59 relative to b‐actin. B, Protein expressions of Gpr41 and Olr59 (top blot) and densitometric analysis normalized to presence of b‐actin (down box & whiskers graph). C, mRNA expressions of Kcnma1 and Kcnmb1 relative to b‐actin. D, Protein expressions of Kcnma1 and Kcnmb1 (top blot) and densitometric analysis normalized to presence of b‐actin (down box & whiskers graph); n = 15 each group (not consanguineous). *P < .05, **P < .01, HY vs CON

Firstly, expressions of SCFAs receptors existed in mesenteric VSMCs were analysed. The mRNA and protein levels of Gpr41 and Olr59 in mesenteric arteries from HY were elevated compared with that from CON (Figure 5A, 5). As shown in Figure 5C, 5, the expressions of BK‐forming alpha subunit (Kcnma1) and main modulatory beta subunit 1 (Kcnmb1) in HY were similar to that in CON.

**Figure 6:**
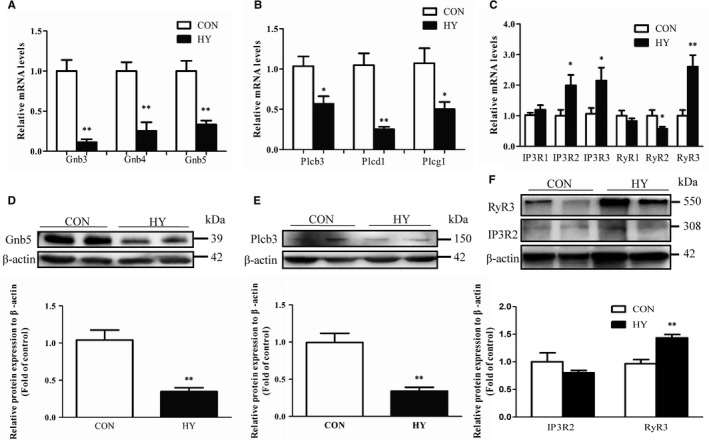
Expressions of G protein b subunits, phospholipase C, IP3R and ryanodine receptor in mesenteric arteries. Relative mRNA expressions of G protein b subunits (Gnb, A), phospholipase C (Plc, B), IP3R (C), and ryanodine receptor (RyR, C). Protein expressions of Gnb5 (D), Plcb3 (E), IP3R2, RyR3 (F), and densitometric analysis normalized to b‐actin; n = 15 each group (not consanguineous). *P < .05, **P < .01, HY vs CON

Expressions of G protein β subunit and PLC in HY were investigated. Among the five types of G protein β subunit in rat, only Gnb3, Gnb4 and Gnb5 were expressed in mesenteric arteries and were down‐regulated in HY (Figure 6A). Consistent with result of mRNA expression, protein expression of Gnb5, the most abundant Gnb, was reduced in HY (Figure 6B). It is known that phospholipase C (PLC), especially PLCβ, acts as a canonical effector of G protein βγ dimer to catalyse the release of diacyl glycerol and inositol 1,4,5‐trisphosphate (IP_3_). As shown in Figure 6C, 6, expressions of PLC family positive in mesenteric arteries were decreased in HY compared to CON. Furthermore, expression levels of IP_3_R and RyR in HY were examined. mRNA expressions of IP_3_R2 and IP_3_R3 rather than IP_3_R1 were elevated significantly in HY (Figure 6E). Although RyR2 in HY mesenteric arteries was decreased, RyR3 (more abundant than RyR2) was raised (Figure 6F). Taken together, reduction of Gnb5 and Plcb3 might be associated with insensitivity of BK to propionate in HY.

## 4 DISCUSSION

Epidemiological studies and investigations on animal models have suggested that the gut and gut microbiome could influence blood pressure. As the unique metabolites of gut microbiome, SCFAs were absorbed from the colon into the bloodstream to modulate host physiology in various tissues.^31^, ^32^ As early as 1990, it was manifested that three SCFAs alone or in combination could cause a concentration‐dependent dilatation in human colonic resistance arteries.^23^ Recently, Pluznick et al^33^ highlighted the contrary effect of SCFAs receptors, Gpr41 and Olr59, on blood pressure. The present study is the first to demonstrate that propionate could significantly evoke opening of BK single channel in mesenteric VSMCs, which can be considered to contribute to vasodilatation. The finding is important for further understanding underlying mechanisms of SCFAs‐mediated vascular regulations and blood pressure. Furthermore, the effect of SCFAs on BK of resistance arteries was tested under a pathologic condition, prenatal hypoxia, which is a typical experimental model for cardiovascular diseases developed in foetal origins.

So far, multiple mechanisms of vasorelaxation by SCFAs have been proposed in several studies, including activation of cGMP system,^34^ increase of cAMP independent of the endothelium 35 and release of endothelium‐derived hyper‐polarization factors.^21^ Whether relaxation of vessels is dependent on intact endothelium is subject to debate, various types of vessels with different distributions of SCFAs receptors might be one of the reasons. In this study, immunohischemistry and RT‐PCR showed Gpr41 and Olr59 appeared in tunica media of mesenteric arteries. VSMCs of small blood vessels in other tissue had been found with positive Olr59 expression.^24^ Therefore, it is plausible that SCFAs regulated VSMC functions directly as Gpr41 and Olr59 ligands, independent of the endothelium.

The important finding in the present study was that sodium propionate could activate BK in VSMCs. Sodium butyrate showed no effects on activities of BK, eliminating the influence of sodium ions and changes in intercellular pH as well as osmotic pressure. In addition, it was proposed that Olr59 responded to propionate rather than butyrate.^24^ Whereas, both of propionate and butyrate activated Gpr41 with a similar potency.^36^ Thus, it was seemed that Olr59 might be associated with the stimulation of BK by propionate. However, the previous work by Pluznick et al had demonstrated Olfr78 (Olr59 in rats) KO mice exhibited hypotension while GPR41 (Gpr41 in rats) KO mice were hypertensive, stating that GPR41 in the endothelium was responsible for mitigation of blood pressure, but binding of SCFAs to Olfr78 in VSMCs increased blood pressure. What is more, Gpr41 is more sensitive to propionate than Olr59. Thus, does Gpr41 or Olr59 play a leading role in propionate‐sensitive BK? Further work was needed to unravel relationship of the two receptors.

**Figure 7:**
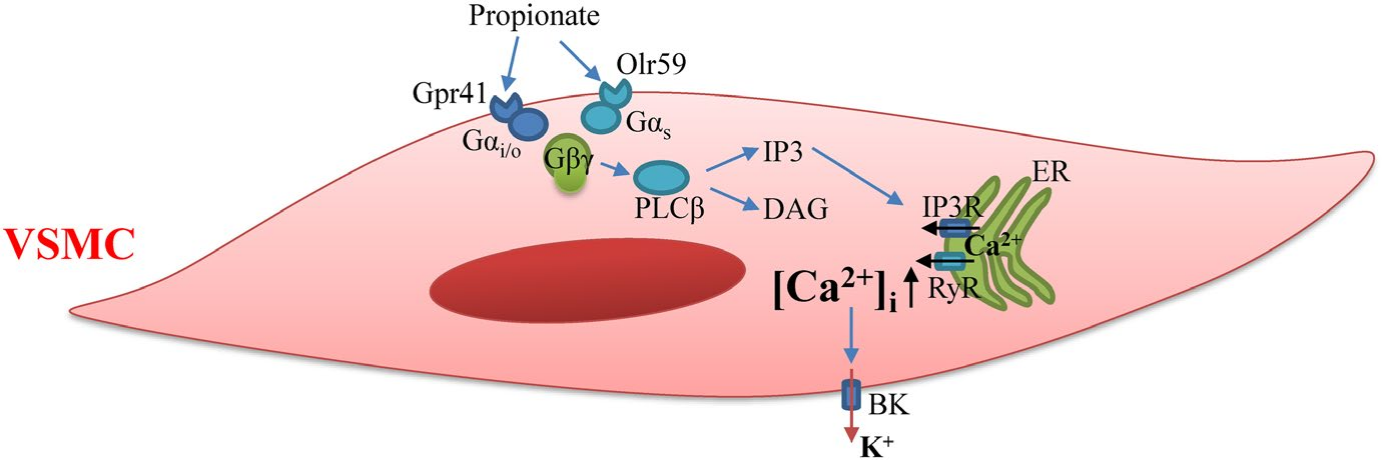
Schematic representation of propionate‐dependent BK activation. Binding of propionate to Gpr41 or Olr59 in VSMCs facilitates the exchange of GDP for GTP on the α subunit of G protein complex, releasing G protein βγ dimer from G protein α subunit. Separated Gβγ dimer activates phospholipase C β (PLCβ), catalysing the formations of inositol 1,4,5‐trisphosphate (IP_3_) and diacylglycerol (DAG). Consequently, IP3R/ryanodine receptor (RyR)‐operated endoplasmic reticulum calcium release is stimulated. Finally, BK channel is evoked by the increase of intercellular calcium ([Ca^2+^]_i_)

Limited information concerning SCFAs receptor signal pathway was available in neurons as well as HEK293 and CHO‐K1 cells expressed recombinant Gpr41.^36^, ^37^ Gpr41 is coupled with Gα_i/o_ to decrease cAMP production, while Olr59 is coupled with Gα_s_ to increase cAMP production. Incomplete block of propionate‐sensitive BK using PTX suggested that there existed PTX‐insensitive G protein responding to propionate. Our study with gallein showed that propionate‐stimulated BK opening depended on Gβγ. Notably, many activation responses related to G protein‐coupled receptors in various cells have been reported to be mediated via Gβγ.^37^, ^38^ Furthermore, endoplasmic reticulum calcium release channel, IP3R, was shown to be necessary to BK activation by propionate. Another channel, RyR, might also be involved. Consequently, it is inferred that propionate activates Gβγ to facilitate endoplasmic reticulum calcium release and afterwards evokes BK single channel (Figure 7). In the process, it is possible that PLCβ links Gβγ and IP_3_R via releasing IP_3_ after stimulated by Gβγ. Therefore, the present study not only demonstrated that propionate significantly evoked opening of BK single channel in VSMCs, but also found the possible pathway or mechanism for its vascular actions.

Notably, the resistance arteries used in the present study were from prenatal hypoxia model. The findings in the offspring vascular systems also offer new meanings for perinatal medicine and prevention medicine, because the data are also helpful for further understanding mechanisms of cardiovascular diseases in developmental origins. Prenatal hypoxia has been shown to impact various tissue and organs in the foetus and offspring, including increasing sensitivity of vasoconstrictions and blood pressure to vasoconstrictors. In the present study, vessel tone responding to PE in HY offspring was enhanced while sensitivity of vasodilation to propionate was reduced by prenatal hypoxia. Consistent with this, propionate‐evoked BK currents in mesenteric VSMCs were depressed by prenatal hypoxia. Taking into account of the key roles in blood pressure regulation of SCFAs, insensitivity of BK and vasorelaxation to propionate might be one of main reasons why risk of hypertension was increased in offspring suffering from prenatal hypoxia. NPo of BK at baseline and expression of BK were not affected by prenatal hypoxia, indicating signal pathway upstream of BK was modulated. Furthermore, down‐regulated Gβ and PLCβ might contribute to insensitivity of BK to propionate in prenatal hypoxia offspring. Increased expression of Gpr41, IP_3_R and RyR3 in HY seemed to be a negative feedback in order to maintain activities of BK. Thus, besides propionate, all stimulations of BK mediated by Gβ and PLCβ might be inhibited by prenatal hypoxia, inducing enhanced vasoconstrictions. Nevertheless, the mechanisms underlying down‐regulation of Gβ and PLCβ in the prenatal hypoxia offspring have not been delineated. Those new information gained may contribute to development of strategies in early prevention of cardiovascular diseases in developmental origins.

## CONFLICT OF INTEREST

No conflicts of interest, financial or otherwise, are declared by the authors.

## AUTHORS' CONTRIBUTIONS

XZ designed the project and wrote the manuscript; ZX revised the manuscript; WZ, XF and YZ performed experiments and analysed data; MS performed statistical analysis; LL, QG and JT assisted with critical paper assessment; PZ and JL assisted with data analysis.

## Data Availability

The data that support the findings of this study are available from the corresponding authors upon reasonable request.
